# Rho GTPases: Big Players in Breast Cancer Initiation, Metastasis and Therapeutic Responses

**DOI:** 10.3390/cells9102167

**Published:** 2020-09-25

**Authors:** Brock Humphries, Zhishan Wang, Chengfeng Yang

**Affiliations:** 1 Center for Molecular Imaging, Department of Radiology, University of Michigan, Ann Arbor, MI 48109, USA; 2 Department of Toxicology and Cancer Biology, College of Medicine, University of Kentucky, Lexington, KY 40536, USA; zhishan.wang@uky.edu

**Keywords:** Rho GTPases, Rho, Rac, Cdc42, breast cancer, metastasis

## Abstract

Rho GTPases, a family of the Ras GTPase superfamily, are key regulators of the actin cytoskeleton. They were originally thought to primarily affect cell migration and invasion; however, recent advances in our understanding of the biology and function of Rho GTPases have demonstrated their diverse roles within the cell, including membrane trafficking, gene transcription, migration, invasion, adhesion, survival and growth. As these processes are critically involved in cancer initiation, metastasis and therapeutic responses, it is not surprising that studies have demonstrated important roles of Rho GTPases in cancer. Although the majority of data indicates an oncogenic role of Rho GTPases, tumor suppressor functions of Rho GTPases have also been revealed, suggesting a context and cell-type specific function for Rho GTPases in cancer. This review aims to summarize recent progresses in our understanding of the regulation and functions of Rho GTPases, specifically in the context of breast cancer. The potential of Rho GTPases as therapeutic targets and prognostic tools for breast cancer patients are also discussed.

## 1. Introduction

Breast cancer is the most common form of cancer diagnosed (about 30% of all new female cancer cases each year) and the second leading cause of cancer-related death (about 15%) in women in the United States [1]. Furthermore, breast cancer is the most invasive type of cancer in women, which highlights the urgent need to better understand the mechanism of breast cancer initiation and metastasis and develop mechanism-based therapeutic strategies for this disease. Signaling networks that function through Rho GTPases have emerged as a field that can provide exciting opportunities for identifying new therapeutic targets and predicting better patient prognosis. Indeed, recent work has demonstrated that breast cancer development, progression and therapeutic responses are underscored by dysregulation of Rho GTPase-dependent signaling pathways. In this review, we focused on recent exciting findings showing the effects of classical Rho GTPase (Rho, Rac and Cdc42) dysregulation on breast cancer initiation and metastasis. We aimed to summarize the specific roles that these Rho GTPases play in each step of breast cancer development and progression, as well as the outlook for their potential to be explored as therapeutic targets and prognosis prediction factors for breast cancer.

## 2. Rho GTPases

**Figure 1:**
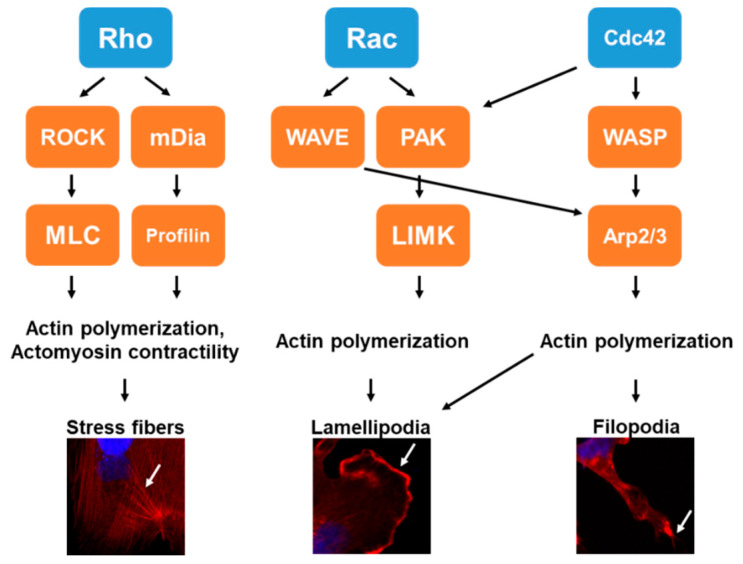
Actin-based structures driven by Rho, Rac and Cdc42 Rho GTPases. General mechanisms and representative images of MDA-MB-231 LM2 breast cancer cells stained with phalloidin (red) and DAPI (4′,6-diamidino-2-phenylindole) (blue) showing the formation of stress fibers (Rho, arrow), lamellipodia (Rac, arrow) and filopodia (Cdc42, arrow) formation. Abbreviations: LIMK, LIM domain kinase; mDia, mammalian diaphanous homolog; MLC, myosin light chain; PAK, p21 (RAC)-activated kinase; ROCK, Rho-associated, coiled-coil-containing protein kinase; WASP, Wiskott-Aldrich syndrome protein; WAVE, WASP family member.

Rho GTPases, a family of the Ras GTPase superfamily, consists of low molecular weight monomeric hydrolyases that are found in all eukaryotic cells. Rho GTPases function as molecular switches by transitioning between a GTP-bound (active or “on” state) and a GDP-bound (inactive or “off” state) conformation (described more in depth by us and others elsewhere [2,3,4,5]). Once activated, Rho GTPases move to the cell membrane or other cellular compartments to interact with downstream effectors such as protein kinases and actin-binding proteins that directly regulate the cytoskeleton as well as influence the dynamics of both the nuclear and cell membranes [6,7]. Through these effects, Rho GTPases regulate many cellular processes, such as membrane trafficking, gene transcription, migration, invasion, adhesion and growth [8,9,10]. Although 20 Rho GTPases have been identified, by far the most well-characterized Rho GTPases are RhoA, Rac1 and Cdc42. Each of these Rho GTPases are central for three separate signal transduction pathways that result in the assembly of distinct actin-based structures [11,12,13,14] (Figure 1). RhoA promotes actin–myosin contractility and activation results in the formation and turnover of stress fibers and focal adhesions, which provide cell–extracellular matrix (ECM) anchoring points. Rac1 activity controls the formation of membrane ruffling and lamellipodia, seen as a large projection at the leading edge of the migrating cell. Finally, Cdc42 signaling results in the formation of filopodia, demonstrated as actin-rich, finger-like projections that protrude from the lamellipodia.

## 3. Regulation of Rho GTPase Activities

**Figure 2:**
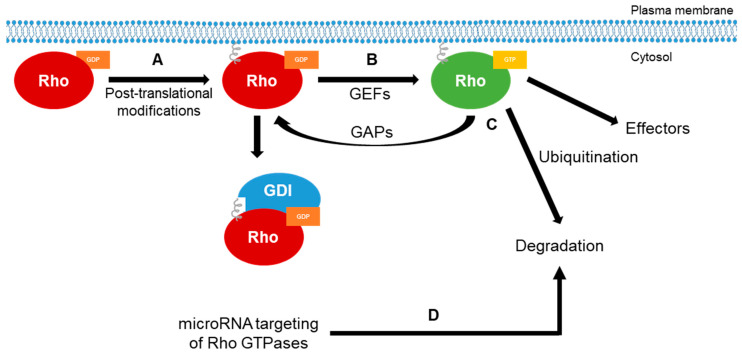
Regulation of Rho GTPases. (**A**) Newly synthesized Rho GTPases are post-translationally modified (such as prenylation and palmitoylation) on the C-terminus to be able to interact with the cellular or an organelle-specific membrane. (**B**) Once lipid modifications are added, Rho GTPases in the guanosine diphosphate (GDP)-bound state (“off” state) can interact with guanine nucleotide exchange factors (GEFs), which facilitate the exchange of GDP for guanosine triphosphate (GTP) and activate the Rho GTPases, or they can also interact with GDP dissociation inhibitors (GDIs), which bind and sequester Rho GTPases in the “off” state. This not only keeps the Rho GTPases from becoming active, but can also protect them from degradation [17]. (**C**) In the GTP-bound conformation, Rho GTPases regulate intracellular signaling cascades through binding and activating downstream effector molecules. This signaling is terminated by the intrinsic GTPase enzymatic reaction, which catalyzes GTP to GDP, and is enhanced by the interaction with GTPase-activating proteins (GAPs). In addition, GTP-bound Rho GTPases can also be sent for degradation by C-terminal ubiquitination or through direct targeting by microRNAs (**D**), terminating signaling. It should be noted that miRNAs bind and negatively regulate Rho GTPase mRNA, decreasing global expression levels of Rho GTPases.

Rho GTPases function as molecular switches that cycle between an inactive guanosine diphosphate (GDP)-bound state and an active guanosine triphosphate (GTP)-bound state, and eukaryotic cells employ three classes of regulators, namely, GTPase-activating proteins (GAPs), guanine nucleotide exchange factors (GEFs) and GDP dissociation inhibitors (GDIs), to control the spatial and temporal activity of Rho GTPases (Figure 2).

Functionally, GAPs promote the catalysis of GTP to GDP to drive GTPase inactivation, GEFs promote the exchange of GDP for GTP which activates GTPases, and GDIs sequester GTPases in the GDP-bound form to keep the GTPase in an inactive conformation. Due to their functions, GEFs are generally thought to have oncogenic functions, while GAPs and GDIs are traditionally thought to have tumor suppressive functions [5,15]. Currently, the number of GAPs and GEFs outnumber the number of GTPases by over 3:1 for each class of regulator, demonstrating that some GAPs and GEFs target the same Rho GTPase. However, research has shown that certain GAPs or GEFs are specific for a single Rho GTPase over others [16,17,18], and this has been reviewed by us [5]. Interestingly, only about half of Rho GTPases are regulated by GAPs, GEFs and GDIs, which are termed classical Rho GTPases. The other Rho GTPases, termed fast-cycling Rho GTPases, are instead regulated through their transcription and are reviewed elsewhere [19].

In addition to these regulators, cells also control the subcellular localization and activity of Rho GTPases through the use of different C-terminal post-translational modifications, including modifications by isoprenoid lipids or palmitate fatty acids, phosphorylation and ubiquitination [20,21,22] (Figure 2). For example, lipid isoprenylation of the C-terminus following Rho GTPase transcription provides a stable plasma membrane anchor [23,24]. Furthermore, Rho GTPases are not limited to a single post-translational modification, as following isoprenylation, Rac1 can also be palmitoylated to help stabilize Rac1 at the actin cytoskeleton [25]. However, these post-translational modifications do not have the same effects for each Rho GTPase because isoprenylation and palmitoylation of RhoB has been shown to induce lysosomal localization and degradation [26]. In addition to post-translational modifications, global expression levels of Rho GTPases can be negatively regulated by microRNAs, resulting in an overall decrease in signaling [24] (Figure 2).

Although some exceptions have been found [27,28], expression of Rho GTPases typically does not change in breast cancer (Table 1), but instead their activity is modified. This can be due to mutations in the Rho GTPase that result in loss of the intrinsic GTPase activity or an increased guanine nucleotide exchange rate [29], or through expression level changes in the GAPs, GEFs or GDIs described above [30]. Nevertheless, changes in Rho GTPase activity underlies breast cancer progression [31,32,33]. Indeed, Rho GTPases have been reported to be involved in almost all steps of breast cancer initiation and metastasis [31].

## 4. Rho GTPases in Cell Transformation, Cancer Stemness and Tumor Formation

Tumor initiation is the process by which normal cells change into malignant cells such that they can form tumors. Spontaneous and environment-induced DNA damage and epigenetic dysregulations likely play important roles which result in permanent changes to the molecular profile of the cell [34,35,36]. Typically, upon experiencing irreparable DNA damage, cells undergo senescence or apoptosis. However, failure to perform these mechanisms can lead to transformation, and ultimately cancer. This is particularly important when considering breast cancer stem cells (BCSCs), which are thought to be a major contributor to breast cancer initiation and progression due to their capability of unlimited self-renewal and the ability to generate all cell types that comprise a tumor [36,37]. Cellular transformation is identified using an anchorage-independent growth assay, also known as a soft agar colony formation assay [38]. This is because the loss of a basement membrane for normal epithelial cells to attach to is required for mRNA and protein synthesis [39]; therefore, the ability to grow without a substrate signifies cellular transformation. Interestingly, recent research has identified an integral role for Rho GTPases in mediating oncogenic transformation.

### 4.1. Rho

Controversial data exist in regard to the role that elevated Rho GTPase activity has in breast cancer cellular transformation [40,41]. For example, Kazerounian et al. used MMTV-PyT (mouse mammary tumor virus-polyoma virus middle T antigen) and MMTV-myc mouse models of spontaneous breast cancer to show that loss of RhoB is critical for cellular transformation in mice, leading to the formation of early mammary lesions [40]. Looking directly at whole-mount staining of mammary fat pads, they found an increased number of early tumor lesions in RhoB^−/−^ (~10 lesions per fat pad) compared with RhoB^+/−^ (~0.5 lesions per fat pad) mice. Furthermore, cells isolated from RhoB^−/−^ tumor lesions displayed enhanced anchorage-independent growth and survival compared with cells from RhoB^+/−^ mice, which did not grow at all. This group found similar results in MDA-MB-231 triple-negative breast cancer (TNBC) cells, where shRNA-mediated silencing of RhoB resulted in increased anchorage-independent growth and a malignant phenotype. It was later determined that RhoB elicits this tumor suppressive function in cell lines by inhibiting basal phosphorylated Akt levels of all three isoforms (Akt1, 2 and 3) through modulation of the level of epidermal growth factor receptor (EGFR) on the cell surface [40].

However, as SUM149 TNBC cells display high levels of RhoC, it is hypothesized that RhoC contributes to cellular transformation in cell lines and partially accounts for its highly invasive phenotype [42]. To test this, RhoC was stably expressed in normal human mammary epithelial (HME) cells. Although stable expression of RhoC did not change cell growth, it did cause HME cells to form significantly more colonies under anchorage-independent growth conditions compared with non-transfected control cells. Unexpectedly, stable expression of RhoC drove a motile and more invasive phenotype through alterations in stress fiber and focal adhesion dynamics. More importantly, stable expression of RhoC increased tumor formation compared with control HME cells in nude mice [42], indicating that RhoC is sufficient for cellular transformation. Interestingly, it was found that reversion of this oncogenic RhoC phenotype, through the use of a farnesyl transferase inhibitor, was accompanied by a concomitant increase in RhoB [43]. This suggests that the reversion of the oncogenic RhoC-mediated phenotype in breast cancer may be mediated by RhoB, and that RhoB and RhoC may have antagonistic roles in TNBC cell lines. Together, these studies suggest that Rho GTPase dysregulations may play an important role in the early stages of carcinogenesis or in cancer initiation.

The acquisition of a stem-like phenotype is thought to be a major driving force giving cells the ability to initiate tumor formation [36,37]. This can be accomplished through EMT (epithelial-mesenchymal transition) and can be defined by gaining the expression of stem cell markers, such as ALDH (aldehyde dehydrogenase) [44]. In this regard, expression of RhoC was found to promote acquisition of a stem-like state [45]. This group found that RhoC expression was positively correlated with ALDH expression, and later identified RhoC as a potential direct regulator of ALDH expression in breast cancer cells. To link this change in stem cell markers to a functional output, this group also demonstrated that knockdown of RhoC reduced tumor formation [45]. Therefore, Rho GTPases can contribute to the acquisition of a stem-like phenotype, and, since they are linked, ALDH activity could be a readout for RhoC activity in breast cancer.

### 4.2. Rac

Work done by Kawazu and colleagues demonstrated key mutations in Rac proteins that promote cellular transformation [28]. This group first isolated cDNAs for *Rac1*, *Rac2* and *Rac3* genes from various breast cancer cell lines and performed Sanger sequencing on them. From this data, they discovered novel Rac1 (Rac1 (P29S), and Rac1 (N92I)) and Rac2 (Rac2(P29L) and Rac2 (P29Q)) somatic mutations, which are also detected in human tumors. To determine the transforming potential of these mutations, this group expressed these mutant proteins in non-tumorigenic MCF10A breast epithelial cells and evaluated anchorage-independent growth in cell culture and tumorigenicity in nude mice [28]. They found that the expression of either the Rac1 or Rac2 mutant protein resulted in enhanced colony formation in soft agar and tumor formation and growth in mice, confirming the transforming capabilities of Rac1 and Rac2 in breast cancer. Mechanistically, they found that these mutations resulted in an increased rate of GDP dissociation, leading to constitutive activation of these proteins. It is interesting to note that the fast-cycling mutations found here in cell lines (P29) occur adjacent to man-made fast-cycling mutants (F28) [46]. This supports previous work showing that the expression of Rho GTPases typically is not changed, but instead their activity is modified in breast cancer. However, it should be noted that other studies have shown that some Rac1 mutations, such as those leading to Rac1 constitutive activation, are rare in human breast tissue [47], suggesting that we should be careful when interpreting results in cell lines.

Using an insertional mutagenesis screen followed by 3′ rapid amplification of cDNA ends (RACE), Goka and Lippman found a different mechanism that leads to Rac1-mediated transformation in breast cancer [48]. From this screen, HACE1 (HECT domain and ankyrin repeat-containing E3 ubiquitin protein ligase 1), an E3 ligase that tags activated Rac1 for proteosomal degradation [49], was identified as a critical suppressor of transformation as HACE1 ablation in MCF12A cells increased anchorage-independent growth in soft agar. Goka and Lippman further found that loss of HACE1 resulted in the accumulation of GTP-bound Rac1 and hyperactive Rac signaling, and stable expression of HACE1 was able to attenuate anchorage-independent growth in these cells by reducing Rac1 activity [48].

Two different studies used a mutant of the Rac and Cdc42 downstream effector, p21-activated kinase (PAK), to study the effects on cellular transformation [50,51]. The expression of a doxycycline-inducible PAK mutant (T423E) that behaves like normal PAK and retains its ability to bind Rac1 and Cdc42 resulted in a significant increase in the ability of MCF-7 breast cancer cells to grow in an anchorage-independent setting [50], and drove hyperplasia in a mouse mammary gland [51]. Looking at the biochemical basis of the ability of this mutant to increase anchorage-independent growth, both groups found an increase in p42/44 MAPK (mitogen-activated protein kinase) and p38 MAPK activation in T423E stably expressing cells treated with doxycycline compared with control cells. Using specific inhibitors of p42/44 and p38, Vadlamudi and colleagues found that MCF-7 breast cancer cells preferentially utilized the p42/44 pathway to increase anchorage-independent growth [50]. It was also found that PAK can regulate ER-driven transcription of target genes [51]. Together, these studies suggest that Rac and Cdc42 drive cellular transformation by activating both of these PAK-mediated pathways.

It has been proposed that induction of cyclins, such as cyclin D1, by growth factors and oncogenes may contribute to cellular transformation [52,53]. To determine whether this is true in breast cancer, Lee et al. utilized an MMTV-neu mouse model to determine the contributions of Rac in Neu-mediated induction of cyclin D1 in MCF-7 breast cancer cells [54]. Using this model, this group found that Neu induced cyclin D1 protein levels up to 12.9-fold relative to the control. The expression of a dominant-negative Rac1 mutant (Rac1(N17) inhibited Neu-induced cyclin D1 promoter activity by 40–50%. Furthermore, this study and others have shown that the dominant-negative Rac1(N17) reduced anchorage-independent growth by about 50% [54,55]. All together, these data suggest that induction of cyclin D1 and cyclin D1-induced cellular transformation involve the activation of Rac.

## 5. Rho GTPases in Primary Tumor Growth and Angiogenesis

### 5.1. Rho GTPases in Primary Tumor Growth

After transformation into a malignant cell, cells typically bypass any cell cycle checkpoints used to suppress growth, resulting in uncontrolled growth and formation of a primary tumor. Microtubule and actin cytoskeleton reorganization during cell division are dependent on Rho GTPases. Therefore, not only have Rho GTPases been shown to be involved in cellular transformation, but studies have shown that they are critically involved in cell cycle and cancer cell proliferation.

#### 5.1.1. Rho in Cell Cycle Progression, Proliferation and Tumor Growth

Initial work demonstrated that increased Rho activity promotes cancer cell proliferation. For example, inhibiting RhoA, B, and C activity by the bacterial protein C3 exotransferase, Liberto et al. discovered that Rho activation is critical for EGF-dependent entry into S-phase of the cell cycle in MCF10A cells [56]. This group found that this is in part due to RhoA-mediated activation of the cyclin D1 promoter. Looking at this more in depth, it was found that EGF-induced DNA synthesis described in S-phase is dependent upon Ras/ERK signaling. Furthermore, they also demonstrated that RhoA synergizes with the Ras(V12) constitutively active mutation to promote entry into S-phase, suggesting that RhoA activity is required for oncogenic Ras to induce cell cycle progression in cells that harbor this mutation [56]. In support of this, Shang and colleagues demonstrated that direct inhibition of RhoA by their drug, Rhosin, inhibited cell growth of MCF-7 breast cancer cells [57]. Lastly, transient degradation of RhoA and RhoC proteins by targeted siRNAs significantly reduced MDA-MB-231 TNBC cell growth in vitro and in mouse models of breast cancer [41]. All together, these data suggest that Rho inhibition reduces cancer proliferation.

Furthermore, RhoB has been shown to positively correlate with proliferation, conflicting with previous studies showing a tumor suppressive function of RhoB in tumor initiation [40,58]. Fritz et al. found that RhoB (as well as RhoA and RhoC) not only has enhanced expression in breast tumors and positively correlated with malignancy, but RhoB also positively correlated with the proliferation index of primary tumors [59]. A more mechanistic study determined that at least in estrogen receptor (ER)-positive breast cancer, RhoB drives proliferation by modulating the expression of the ER, leading to changes in the cellular response to estrogen, in multiple ER-positive breast cancer cell lines [60].

However, recent work in our lab has shown an important role for RhoA activation for inhibiting breast cancer proliferation [61]. Using MDA-MB-231 and SUM159 TNBC cells that stably expressed miR-200b, we were able to show that stable expression of miR-200b enhanced RhoA activation through direct targeting of ARHGAP18, a RhoA-specific GAP. We demonstrated that increased RhoA activation through decreased ARHGAP18 expression caused a decrease in cell proliferation compared with control cells that have decreased RhoA activity in breast cancer.

#### 5.1.2. Rac and Cdc42 in Cell Cycle Progression, Proliferation and Tumor Growth

Yang and colleagues found that Rac1 is an essential mediator of heregulin β1 (HRG)-induced cell proliferation [62]. In addition to promoting cellular transformation, this group also found that HRG-induction of cyclin D1 and p21^Cip1^ resulted in an increase in cell proliferation. To determine if Rac1 was involved in this process, they next either expressed a Rac-specific GAP (β2-chimearin), the dominant-negative Rac1(N17) mutant, or depleted Rac1 by RNAi (RNA interference). All three methods resulted in a reduction of cyclin D1 and p21^Cip1^ expression levels, suggesting that Rac1 is essential for HRG-mediated cell cycle progression and proliferation [62]. In support of this data, this group also found that wild type β2-chimaerin, but not mutated forms of β2-chimaerin lacking Rac-GAP activity, significantly reduces cell cycle progression through a reduction of cyclin D1 [63], suggesting a Rac-dependent effect on cell growth. Furthermore, the Rac1(N17) dominant-negative mutant also showed that Rac1 inhibits cell proliferation of a highly aggressive and invasive MTLn3 breast cancer cell line [55].

In addition to Rac1 described above, Rac3 has also been shown to control cell proliferation [64]. Comparing several breast cancer cell lines, Mira et al. found that the cell lines that grew the most under low serum conditions displayed more active Rac3 than slowly growing cells. Consistent with this cell line data, hyperactive Rac3 was also found in human metastatic breast cancer tissues. The expression of a constitutively active Rac3 mutant (Rac3(V12)) in HMEC184 normal mammary epithelial cells, which endogenously express low levels of active Rac3, caused increased proliferation as assessed by a BrdU (Bromodeoxyuridine) incorporation assay. Mechanistically, it was found that Rac3 promotes cell proliferation through the p21-activating kinase (PAK)-JNK (c-Jun N-terminal kinase) cascade [64].

Research has also shown that Cdc42 is integral for EGF (epidermal growth factor)-mediated cell proliferation in breast cancer [65]. Comparing MDA-MB-231 and BT20 TNBC cells with MCF-7 estrogen-positive (ER+) breast cancer cells, Hirsch et al. found that the TNBC cells expressed > 3-fold higher levels of activated Cdc42 compared with MCF-7 cells, and this increase positively correlated with expression levels of EGFR. Although silencing of Cdc42 through RNAi resulted in inhibition of TNBC cell proliferation, they found no change in the DNA content in cells, which suggests that the effects on proliferation were due to inactivation of Cdc42. Interestingly, this group found that silencing Cdc42 significantly reduced levels of EGFR on the surface of cells, and that expression of a c-Cbl-N480 mutant, which does not associate with active Cdc42 but still binds and ubiquitinates EGFR, blocks Cdc42-induced proliferation [65]. These data together suggest that Cdc42 is critical for EGF-induced breast cancer cell proliferation.

### 5.2. Rho GTPases in Angiogenesis

As tumors grow, the cells at the center of the tumor are subjected to hypoxic conditions [66]. Therefore, for metastatic spread to occur, growth and integration of the vascular network are important. Tumors overcome this hypoxic barrier by hijacking their surrounding cells to initiate angiogenesis. This allows new blood vessels to form within the tumor, alleviates nutrient deficiency and promotes metastasis. As Rho GTPases function within specific vascular cell types, and some Rho GTPases are also expressed in diseased vessels [67], recent studies have shown that Rho GTPases are important for angiogenesis.

#### 5.2.1. Rho

In addition to promoting cellular transformation and tumor formation described above, Kazerounian and colleagues found that RhoB also contributes to angiogenesis [40]. Analyzing human tumor and normal tissues, they found that RhoB is highly expressed in the tumor-associated blood vessels compared with blood vessels of adjacent normal tissue, suggesting that RhoB is important for tumor vasculature. This data was further confirmed in their mouse model where tumors from RhoB^-/-^ mice displayed less CD31, an endothelial cell marker, and displayed a general defect in angiogenic sprouting.

Two separate studies from the same group have shown that RhoC regulates angiogenesis in an inflammatory and TNBC cell line [68,69]. An ELISA analysis on cell-conditioned media from HME cells with and without stable expression of RhoC demonstrated that cells that stably express RhoC produce significantly more angiogenic factors compared with cells that do not stably express RhoC. More specifically, this group found that RhoC-expressing cells made around 10-fold more vascular endothelial growth factor (VEGF) and basic fibroblast growth factor (bFGF), as well as about 5-fold more interleukin-6 (IL-6) and interleukin-8 (IL-8). HME cells stably expressing RhoC also produced these angiogenic factors at levels comparable to parental SUM149 TNBC cells, which express high levels of endogenous RhoC. To test the functionality of the angiogenic factors, van Golen et al. used a rat aortic ring assay, which showed that expression of RhoC promoted microvessel outgrowth. The inhibition of RhoA/B/C by C3 exotransferase reduced the production of VEGF, bFGF, IL-6 and IL-8, suggesting that production of these angiogenic factors was Rho-dependent.

#### 5.2.2. Rac and Cdc42

To determine the effects of Rac1 and Cdc42 on angiogenesis, Ma and colleagues incubated human umbilical vein endothelial cells (HUVECs) with conditioned media from MCF-7 cells that stably expressed either the Rac1(V12) or Cdc42(L61) constitutively active mutant [70]. They found that the conditioned media from either constitutively active mutant induced the formation of significantly more capillary tubes, which is a precursor to and surrogate for blood vessel formation, compared with the control. Targeted RNAi experiments showed that inhibition of Rac1 and Cdc42 resulted in the concomitant reduction of VEGF, and overexpression of either the Rac1(V12) or Cdc42(L61) mutant drove an increase in VEGF expression. Interestingly, it was later determined that p53 was responsible for mediating this Rac1- and Cdc42-mediated induction of VEGF [70].

Using a Rac-specific inhibitor, another study has also identified a critical role for Rac in angiogenesis. As they had previously found that mammary tumors treated with their Rac-specific inhibitor (EHop-016, which inhibits Rac activity through inhibiting the oncogenic GEF Vav [71]) showed an 85% decrease in surface blood vessels compared with vehicle-treated tumors [72], Castillo-Picardo and colleagues wanted to determine if EHop-016 inhibited angiogenesis. To this extent, treating HUVECs with EHop-016, they found that the inhibitor inhibited capillary tube formation in vitro. Together, these data suggest that Rac and Cdc42 activity is important for angiogenesis in breast cancer.

## 6. Rho GTPases in Breast Cancer Metastasis

Metastasis is a complex, multi-step process by which a cancer cell moves away from the primary tumor, resulting in the development of a secondary (termed metastatic) tumor at a distant organ separate from the primary site of cancer. As metastasis is a highly inefficient process [73], the cells that accomplish this have likely undergone extensive genetic and epigenetic changes that benefit the survival, growth, migration and invasive capabilities of the cell. In general, metastasis is broken down into six major steps [74,75]: (1) primary tumor growth and angiogenesis, (2) nearby tissue invasion and migration away from the primary tumor, (3) intravasation into the blood and lymphatic systems, (4) survival and circularization within these systems, (5) extravasation out of the blood and lymphatic systems and (6) outgrowth and colonization of the secondary site. Each one of these steps represents an important barrier that tumor cells must overcome in order to successfully metastasize.

The current knowledgebase of research into Rho GTPases demonstrates that they can affect each step of the metastatic cascade. Therefore, this section will summarize this work with respect to the six major steps described above.

### 6.1. Rho GTPases in Migration and Invasion

A critical property that cells must acquire in order to successfully metastasize is the ability to move away from the primary tumor site. Cells migrate in vitro and in vivo as either single cells or as a collective (sheets or groups) [76], which is driven by the dynamic formation and disassembly of actin-based structures. Rho GTPases are critical regulators of the actin cytoskeleton, and thus have been shown to be critical mediators of cancer cell migration and invasion [77].

#### 6.1.1. Rho

Studies with RhoA have demonstrated controversial effects on breast cancer cell migration. Shang and colleagues developed an inhibitor that specifically binds RhoA and inhibits GEF-mediated activation [57]. Using this inhibitor, this group found that inhibition of RhoA significantly decreased MCF-7 cell migration and invasion as determined by a transwell assay. In a separate study utilizing a FRET (Fluorescence resonance energy transfer) reporter that can allow for real-time quantification of RhoA, enhanced RhoA activity was found in the invasive PyMT-induced breast tumors relative to control tumors [78]. Similarly, two further studies demonstrated that silencing RhoA by RNAi reduced the migration and invasion of MDA-MB-231 [41] and SUM149 [79] TNBC cells.

However, we and others have demonstrated that in MDA-MB-231 and BT20 cells, inhibition of RhoA actually promotes migration and invasion, ultimately leading to successful metastasis. In addition to inhibiting proliferation described above, enhanced activation of RhoA by stable expression of miR-200b also resulted in decreased migration as assayed in a wound healing assay [61]. The re-expression of ARHGAP18, a RhoA-specific GAP, was able to overcome the inhibitory effects of RhoA activation on migration. In support of this data, Kalpana et al. demonstrated that knockdown of RhoA increased Matrigel invasion in two TNBC cell lines and 4T1 mouse mammary cells [80]. Furthermore, the expression of a dominant-negative RhoA(T19N) promoted, and the constitutively active RhoA(Q63L) decreased, 4T1 cell invasion. Lastly, this group demonstrated that RhoA expression was sufficient to inhibit 4T1 invasion into the sentinel lymph nodes of BALB/c mice partially through modulating CCL5-CCR5 and CXCL12-CXCR4 chemokine signaling. All together, these studies suggest contradicting effects, and may point to a more context-dependent role for RhoA on breast cancer motility and invasion.

Similar to RhoA, RhoB effects on breast cancer migration and invasion are complex. In MDA-MB-231 TNBC cells, Ju et al. found a decrease in migratory velocity, persistence and displacement of cells that stably expressed RhoB or complete RhoB knockout cells in a 3D collagen matrix [81]. A decrease or increase of RhoB also significantly reduced spheroid invasion compared with wild-type (WT) control spheroids under hypoxia conditions. However, in contrast to migration and invasion, they found that reduced RhoB did not affect invasion into lymph nodes, whereas RhoB stable expression did reduce lymph node invasion [81]. These data suggest that either increased or decreased RhoB expression has significant effects on breast cancer migration and invasion.

In contrast to RhoA and RhoB, research is more conclusive on the enhancing effects of RhoC on breast cancer migration and invasion. High levels of endogenous RhoC found in SUM149 TNBC cells or stable expression of RhoC in HME cells resulted in enhanced cell migration and invasion, and these oncogenic effects of RhoA/B/C were abrogated by treatment with the C3 exoenzyme Rho inhibitor [69]. Another study found that Akt1 (Protein kinase B) is likely responsible for the enhanced migration in SUM149 TNBC cells because it directly phosphorylates RhoC [82]. In further support of the oncogenic effect of RhoC, siRNA silencing of RhoC decreased breast cancer migration and invasion [41,79,83].

#### 6.1.2. Rac and Cdc42

Feng and colleagues found that Rac1 activation is critical for RASAL2 (RAS protein activator like 2)-mediated TNBC invasion [84]. This group profiled global miRNA expression in the aggressive MDA-MB-231 and BT549 TNBC cell lines. They found that miR-203 was significantly decreased in these cell lines, and further analysis confirmed that RASAL2, a Ras GAP, was a direct target of miR-203. In contrast to its role in luminal ER-positive breast cancer, patient data analysis showed that RASAL2 expression correlated with worse prognosis in TNBC. It was demonstrated that the oncogenic activity of RASAL2 was mediated by Rac1 activation, and that Rac1 activation promoted invasion [84]. Mechanistically, it was determined that Rac1 activation was increased in these cells because RASAL2 antagonizes the Rho GAP, ARHGAP24, suggesting a higher complexity to Rho GTPase activity regulation by GAPs, GEFs and GDIs.

Many studies have used or identified new Rac inhibitors to study the mechanistic effects of Rac inhibition on migration and invasion. To this extent, Katz et al. found that Rac1 and Rac1b, a constitutively active mutant Rac1 isoform that only differs by 19 amino acids in frame insertion, are highly expressed and active in primary breast cancers cultured for a long period of time in a collagen-based matrix [85]. The treatment of these explants with EHT1864, a pan-Rac inhibitor, blocked invasion into the surrounding collagen of the culture system. It was found that this was accomplished through a Rac1-STAT3-dependent mechanism. ZINC69391 was also identified as an inhibitor for Rac1 as it blocks the ability of Rac1 to interact with GEFs [86]. Further investigation with the inhibitor showed that preventing Rac1 activation by ZINC69391 reduced EGF-induced actin reorganization and reduced cell migration as assayed by wound healing in MDA-MB-231 and F3II breast cancer cells. De and colleagues used NSC23766 and W56, other Rac1 inhibitors, in various TNBC cells to demonstrate that Rac1 activation was necessary for Wnt3A (Wnt family member 3A)- and fibronectin-stimulated migration and invasion [87]. Lastly, Humphries-Bickley et al. synthesized a Rac/Cdc42 dual inhibitor, MBQ-127, which targets the shared downstream effector (p21-activated kinase (PAK), Figure 1) and is a much more potent inhibitor than other specific inhibitors (such as EHop-016) [88]. The inhibition of Rac and Cdc42 by MBQ-127 resulted in a significant reduction in migration as well as invasion. All together, these data show that enhanced Rac1 activation promotes breast cancer migration and invasion. However, studies have shown that some of these Rac inhibitors, in particular NSC23766 and EHT1864, have some critical Rac-independent effects on normal cells in mice at concentrations similar to those used in cancer studies [89]. Therefore, data from studies using these inhibitors should be analyzed cautiously.

Research in our lab utilizing microRNAs supports the oncogenic effect of Rac1 on breast cancer cell migration. We found that stable expression of either miR-200b [90] or miR-205 [91] in two TNBC cell lines, MDA-MB-231 and SUM159, significantly reduced Rac1 activation. We later determined that this reduction of Rac1 activation resulted in a concurrent decrease in cell migration compared with control cells as defined by a wound healing and transwell cell migration assay. We also found that miR-205 expression inhibited invasion through Matrigel [91], further confirming the oncogenic role of Rac1 in breast cancer cell migration and invasion.

In support of Rac1 function in breast cancer, studies have also shown that Rac3 activation promotes invasion. Work specifically with invadopodia, actin-rich protrusions that help degrade the ECM [92], have demonstrated that Rac3 activation is critical for their formation and maturation, and that Rac3 activation at invadopodia is mediated by Vav2 and βPIX (ARHGEF7) Rho GEFs in TNBC cells [93,94]. However, Rac3-mediated invasion may be specific for TNBC as reduction of Rac3 in MCF-7 cells, a less aggressive and luminal breast cancer cell line, did not have any significant effects on parameters of aggressiveness [95].

Research has shown that Cdc42 activation promotes breast cancer cell migration. As disruption of epithelial architecture is a hallmark of breast cancer, Bray and colleagues developed a TetO-Cdc42-overexpressing mouse model to study the effects of Cdc42 on epithelial structure [96]. Using this model, this group found that the expression of Cdc42 drove increased mammary gland branching. Isolating epithelial cells from the mammary gland, Cdc42-overexpressing mammary gland cells were found to migrate and invade significantly more than matched control cells, and this was accompanied by an increase in MAPK signaling. In support of this, it was also found that Cdc42 activation promoted the ability of breast cancer cells to close a wound and migrate in a Boyden chamber [65]. However, the expression of wild-type Cdc42 was found to have no significant effect on the ability of cancer cells to migrate across collagen [97], suggesting context and substrate specificity for Cdc42 effects on motility. Altogether, these data demonstrate the pro-migratory effects of Rac and Cdc42 activation in breast cancer cells.

### 6.2. Rho GTPases in Intravasation and Survival in Circulation

To successfully metastasize, a cell must enter the circulation, a process termed intravasation, and survive. Cells that successfully enter the circulation are termed circulating tumor cells (CTCs). Not only are the properties of the vasculature, including vessel density and diameter, important for intravasation, but also gene expression changes within the cell greatly attribute to a cell infiltrating into the blood and lymphatic systems. For example, Notch signaling [98], tumor-associated macrophages (TAMs) [99] and transforming growth factor β (TGFβ) signaling [100] have been shown to enhance intravasation. Additionally, new blood vessels formed in the primary tumor through angiogenesis are leaky and can also facilitate angiogenesis [101,102]. Limited information exists about the role of Rho GTPases in intravasation, but the work done suggests that Rho GTPase activity promotes intravasation and survival in circulation. Reymond and colleagues identified Cdc42 as a critical regulator of breast cancer cell intravasation, as RNAi targeting of Cdc42 significantly inhibited the ability of MDA-MB-231 cells to migrate and invade through a layer of endothelial cells [103]. Additionally, dominant-negative mutants of RhoA (RhoA(N19)), Rac1 (Rac1(N17)) and Cdc42 (Cdc42(N17)) each significantly reduced the number of cells that were found in the blood stream after a subcutaneous injection of MTLn3 breast cancer cells into nude mice compared with wild-type Rho GTPases [55], suggesting that Rho GTPases are not only critical for intravasation, but also for survival in circulation.

Along these lines, once cells have successfully intravasated into circulation they need to adapt to a harsh environment to survive. To facilitate survival, CTCs often reprogram certain cellular processes such as apoptosis and anoikis to survive. In this regard, the expression of geranylgeranylated RhoB, one of the major prenyl modifications critical for Rho GTPase function, was found to promote apoptosis of breast cancer cells [104]. In contrast, loss of Rac1 activation through an inhibitor (NSC23766) induced apoptosis, and the expression of a dominant-active Rac1 mutant (Rac1(V12)) was able to rescue the effects of the inhibitor [105]. Therefore, more research is needed to identify the effects of specific Rho GTPases in breast cancer apoptosis.

### 6.3. Rho GTPases in Extravasation and Metastatic Colonization

The final steps in the metastatic cascade require cancer cells to arrest in circulation, extravasate from the circulation and proliferate to colonize a distant organ. The number of CTCs far exceeds the number of metastatic foci that develop [106], suggesting a highly inefficient process. However, cells can alter cytoskeletal components to enhance migration and invasion to the distant organ and increase the efficiency of metastasis [107]. Therefore, Rho GTPase activity is important for effective colonization by breast cancer cells.

#### 6.3.1. RhoA

In support of actin cytoskeleton rearrangements important for metastasis, studies have found that activation of RhoA is critical for colonization [55,108]. Utilizing tail vein injection, the knockdown of RhoA, either through RNAi or expression of a dominant-negative mutant, reduced or blocked MDA-MB-231 metastatic lung colonization, respectively. However, recently it was found that RhoA knockdown had no significant effect on metastatic lung colonization of breast cancer cells in a syngeneic mouse model of breast cancer [80]. Discrepancies between the outcomes of these studies may arise from the different cells used in the assay or the method used to reduce RhoA activation, or due to the difference in time points at which the measurements were taken. Nonetheless, more research is needed to discern the function of RhoA activity on metastatic colonization.

#### 6.3.2. Rac1 and Cdc42

In addition to reducing migration and invasion as described above, the Rac1-specific inhibitor ZINC69391 also inhibited lung colonization in a syngeneic animal model [86]. To specifically measure extravasation and metastatic colonization, F3II breast cancer cells were injected into the tail vein of BALB/c mice. This group found that daily treatment of mice with ZINC69391 significantly reduced the formation and size of metastatic lung colonies. In support of this, the expression of the dominant-negative Rac1 mutant (Rac1(N17)) also completely blocked metastatic lung colonization [55]. Similar to studies with Rac1, inhibition of Cdc42 activation by RNAi or expression of a dominant-negative mutant reduced the ability of breast cancer cells to form lung metastatic foci [55,103]. Altogether, this data suggests that the activation of Rac1 and Cdc42 is critical for breast cancer extravasation and metastatic colonization, and that ZINC69391 may provide a promising therapeutic option for aggressive breast cancer.

## 7. Rho GTPases in Breast Cancer Therapy Resistance

One of the most challenging barriers to successfully treating cancer is the development of chemoresistance or radioresistance. Developing either of these resistances not only promotes metastasis, but they are also thought to be primary causes of disease recurrence. Mechanisms that contribute to these resistances include, but are not limited to, (1) inadequate delivery of the chemotherapeutic, (2) undergoing epithelial-to-mesenchymal transition (EMT, a developmental program where an epithelial cell acquires mesenchymal-like characteristics such as increased migration and invasion) and (3) activation of oncogenic and inhibition of tumor suppressive signaling pathways [109,110,111,112]. Focusing on cellular reprogramming, cells effectively evade drugs by altering genes involved in resistance, and as such studies have shown that altering Rho GTPase activation plays a role in both chemoresistance and radioresistance. One of the major mechanisms underlying chemoresistance is acquisition of a stem-like phenotype, and the effects of Rho GTPases on the acquisition of a stem-like phenotype is discussed above. Therefore, this section will discuss the effects of Rho GTPases on chemoresistance and radioresistance.

In support of RhoC as a mediator of chemoresistance, Kawata and colleagues separately found that RhoC protein expression was significantly higher in breast tumor tissue after chemotherapy compared with tumor tissue taken before therapy [113]. To discern whether RhoC is important for response to therapy, they tested whether this also happened in culture. Indeed, treatment of MCF-7 breast cancer cells with the chemotherapeutic, etoposide, resulted in increased RhoC expression, and stable expression of a constitutively active RhoC resulted in the induction of an EMT-like phenotype characterized by a reduction of membrane-expressed E-cadherin. As described above, RhoC expression is positively correlated with the expression of ALDH, a breast cancer stem cell marker, and is a potential direct regulator of ALDH expression in breast cancer cells [45]. Since ALDH has been shown to confer cellular resistance to a variety of cytotoxic drugs used for breast cancer, such as cyclophosphamide and its active derivative [114,115], doxorubicin [116] and cisplatin [116,117], this offers a potential mechanism for how RhoC mediates chemoresistance in breast cancer cells.

Zhao and colleagues found that Rac1 is highly activated in trastuzumab-resistant SKBR3 breast cancer cells [118]. To confirm whether Rac1 plays an important role in trastuzumab-resistant cells, this group inhibited Rac1 activity using NSC23766 and then tested the sensitivity to trastuzumab. While trastuzumab or NSC23766 alone decreased cell viability, combined treatment decreased cell viability significantly more than either of the treatments alone. To further confirm the role of Rac1 in trastuzumab resistance, they treated cells with an siRNA directed against Tiam1, a Rac1 GEF, and found results similar to that of NSC23766 treatment on cell viability [118]. Together, these results demonstrate that Rac1 activation plays a critical role in regulating trastuzumab resistance in breast cancer.

In addition to chemotherapy, patients are often treated with small doses of targeted ionizing radiation (IR), also known as radiotherapy, to kill breast cancer cells. Two separate studies demonstrated that subjecting breast cancer cells to IR resulted in the immediate increase of Rac1 [119,120]. To confirm the role of Rac1 in radioresistance, they found that inhibition of Rac1 using a specific inhibitor (NSC23766), a dominant-negative Rac1(N17) mutant, or RNAi drove cell death in response to IR, suggesting that a decrease in Rac1 may help sensitize cells to radiotherapy.

## 8. Use of Rho GTPases as a Prognostic Tool for Breast Cancer

Rho GTPases have been shown to be difficult to develop drugs against due to their structure, their affinity for GTP and GDP and the high concentration of GTP inside cells (described elsewhere [121,122,123]). In addition to their structure, the fact that Rho GTPases are critically involved in numerous processes also adds an additional layer of complexity to successfully targeting Rho GTPases in cancer [121,124]. Therefore, it is a more reasonable approach to target the activators of Rho GTPases [15,123]. Alternatively, a potential effective clinic use for Rho GTPases is as prognostic biomarkers. This is because identifying biomarkers that can serve as an early detection method or as a predictive tool is essential for better patient prognosis. Research on the efficacy of using Rho GTPases as prognostic tools in breast cancer is somewhat limited, where current prognostic studies focus either on the expression levels of Rho GTPase regulators (GAPs, GEFs or GDIs) or have found expression changes in the Rho GTPases themselves. Overall, the literature suggests that Rho GTPase expression and activity are positively associated with breast tumor progression and metastasis.

Studies have shown the potential of Rho GTPases themselves in patient prognosis. A study by Fritz et al. found that the protein expression of Rho, but not Rac or Cdc42, varied across histological tumor grade [27]. Specifically, grade I tumors displayed low protein expression of RhoA, B and C, whereas their expression was increased in grade III tumors. Rho GTPase protein expression also positively correlated with the expression of Ki-67, a marker of proliferation, in human breast samples. Interestingly, this correlation was not found with Rho GTPase mRNA, suggesting that the change in expression is not regulated on the mRNA level. In support of this, RhoC protein was found to be exclusively expressed in invasive breast carcinomas and not in normal breast, atypical intraductal hyperplasia or ductal carcinoma in situ, and increased levels of RhoC protein positively correlated with node-positive tumors [125,126,127]. The Rac1b isoform was also found to be increased in breast tumors compared with the surround stroma, and MMP3, a known upstream activator of Rac1b, was found to positively correlate with distant metastasis-free (DMFS) and higher grade luminal and basal subtypes of breast cancer [128].

In terms of Rho GTPase regulators, Aleskandarany and colleagues found that the expression of ARHGAP18, a RhoA-specific GAP, is associated with improved prognosis for patients with breast cancer [129]. Interestingly, they found that if ARHGAP18 was expressed in both the cytoplasm and nucleus, it was associated with improved prognosis. However, if ARHGAP18 was only expressed in either the nucleus or cytoplasm, it was positively correlated with lymphovascular invasion and higher grade, respectively, in breast cancer [129]. This suggests that cellular localization is an important factor that needs to be taken into account with Rho GTPase regulators and patient prognosis. In addition to finding the correlation of enhanced RhoC protein levels with node-positive breast tumors, Jiang et al. also found that expression levels of RhoGDIɣ was inversely correlated with spread to lymph nodes and grade [126]. Interestingly, this study also found no change in the expression levels of RhoA between tumor and normal tissues, which contradicts earlier studies showing differences in RhoA [27]. Similarly, high levels of three Rho GTPase activators (GEFs), Trio (trio Rho guanine nucleotide exchange factor), Vav1 (vav guanine nucleotide exchange factor 1) and TIAM1 (T cell lymphoma invasion and metastasis 1), were found in breast tumors compared with normal background breast tissue, and positively correlated with a poor prognostic index [130]. More specifically, they also found that the level of TIAM-1 was significantly higher in tumor tissue from patients who died from breast cancer compared with those who survived. The finding that these GEFs are found differentially expressed between breast cancer and normal tissues supports other studies that also found expression changes of other Rho GTPase regulators in breast cancer [30,131,132,133]. Together, these data suggest that increased protein expression of certain Rho GTPases and Rho GTPase activators is correlated with worse patient prognosis, and that Rho GTPases may be promising prognostic tools in the clinic. However, due to the complexity of Rho GTPase regulation and the controversial function of Rho GTPases in breast cancer, more research is needed to look into the role of Rho GTPases as a prognostic marker or biomarker.

## 9. Conclusions and Future Perspectives

**Figure 3:**
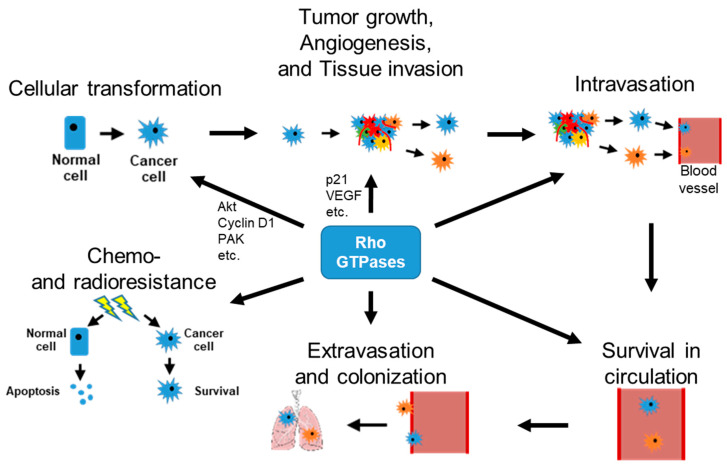
Effects of Rho GTPases on steps of cancer initiation and metastatic progression. Although some studies have shown conflicting data (specifically in the case of Rho), the literature generally agrees that increased activation of Rho GTPases results in breast cancer initiation, metastatic progression, chemoresistance and radioresistance.

Studies on Rho GTPases in breast cancer have greatly enhanced our knowledge about the critical roles they play in breast cancer development and metastasis. Although contradicting data do exist, the literature generally supports the notion that Rho GTPase activation drives breast cancer progression by affecting each step of the metastatic cascade (Figure 3).

Future research will help to better identify the mechanisms underlying Rho GTPase effects on breast cancer initiation, therapy resistance and metastasis. Due to their well-known effects on the actin cytoskeleton, much of the work surrounding Rho GTPases focuses on the effects on migration and invasion. However, the other steps of the metastatic cascade are understudied, and therefore, studies to determine the effects of Rho GTPases on these steps and processes are needed. Furthermore, research will need to better identify the context in which each Rho GTPase acts as an oncogene or tumor suppressor in breast cancer. This is particularly necessary for RhoA, where data support both oncogenic and tumor suppressive roles in breast cancer progression. Since studies have identified loss-of-function RhoA mutations in other types of cancers [134,135,136,137,138], we argue that evidence supports that wild-type RhoA functions as a tumor suppressor. As the inhibition of RhoA in the methods described above does not discriminate between wild-type and mutant forms, this may lead to the oncogenic function demonstrated in their studies. This is also exacerbated by the fact that current studies with Rho GTPases utilize methods that result in expression changes of the Rho GTPase, but expression changes of Rho GTPases are not typically found in human tumors and cancer tissues. In any case, more research is needed to support or refute this hypothesis.

In addition to understanding the context-dependent roles of Rho GTPases in breast cancer initiation and progression, the identification of localization-specific effects of Rho GTPase regulators warrants further investigation. This work found that either nuclear or cytoplasmic localization of a Rho GAP promoted cancer progression, while expression of this GAP in both the nucleus and cytoplasm correlated with better patient prognosis [129]. As nuclear localization and cytoplasmic localization have been reported for other Rho GAPs [139,140], this may be a common mechanism for regulating Rho GTPase activation in breast and other cancers. While this will help in our understanding of localization-dependent regulation of Rho GTPase activity, it may also offer a prognostic tool for predicting the prognosis of breast cancer.

Lastly, the vast majority of the work on Rho GTPases on breast cancer initiation and progression focuses on the three most prominent members, RhoA, Rac1 and Cdc42. Future work will need to address the effects of other classical and non-classical Rho GTPases, as these Rho GTPases may also offer attractive targets and therapeutic options for breast cancer. Overall, Rho GTPases in breast cancer provide new challenges and opportunities for the development of new general and personalized therapeutic strategies for breast cancer.

## Figures and Tables

**Table 1:** Mutational profiles in breast cancer of the classical Rho GTPases discussed in this review. All mutational analyses were taken from cBioPortal for invasive breast cancer (1084 total cases).

Rho GTPase	Percentage (%) Mutated in Invasive Breast Cancer	Most Prominent Mutation Type (Percentage (%) of Total Cases)
RhoA	1.38	Deletion (0.46)
RhoB	0.37	Amplification (0.18)
RhoC	0.83	Amplification (0.28)
Rac1	0.46	Amplification (0.37)
Rac2	0.37	Amplification (0.37)
Rac3	4.15	Amplification (3.69)
Cdc42	0.83	Deletion (0.37)
